# Supplementary material for: Content-rich biological network constructed by mining PubMed abstracts
Source: BMC Bioinformatics. 2004 Oct 8;5:147. doi: 10.1186/1471-2105-5-147 (PMC528731; doi:10.1186/1471-2105-5-147)
Supplement: Additional File 2 — The original results of the above study (non-essential files are deleted to keep the file size under the limit set by BMC bioinformatics). [file 1471-2105-5-147-S2.bz2 › chilibotAdditionalFile2/dip10/74ID7513045E283/html/CD5_LCK.html]

 


 **CD5** and **LCK** 
  
Found 17 abstracts in PubMed, retrieved 10.  
 

 What does Google say? 
 PDF only 
| .edu only 

---

**Interactive relationship** (e.g. stimulation, inhibition, etc)

**Neutral relationship**- The analysis of  **Lck**  and CD3 deficient Jurkat cells shows that tyrosine phosphorylation of  **CD5**  requires  **Lck**  activity.  Ref: 11298344 Eur J Immunol, 2001
- Using a T cell line deficient in the tyrosine kinase p56  **lck**   [ **LCK** ]  and the same cell line reconstituted with this kinase, we show that p56  **lck**   [ **LCK** ]  expression is required for efficient  **CD5**  tyrosine phosphorylation.  Ref: 11157848 Int Immunol, 2001
- We have recently shown that B 1 cells, which are characterized by  **CD5**  surface expression, specifically exhibit significant levels of the tyrosine kinase  **Lck**  in man.  Ref: 12645942 Eur J Immunol, 2003
- Here we show that pMHC contact modulates the expression of  **CD5**  by naive CD4 T cells in a process that requires the continued expression of p56  **lck**   [ **LCK** ] .  Ref: 11696591 J Exp Med, 2001
- induction of  **CD5**  surface expression on DN thymocytes requires engagement of the pre TCR and is dependent upon the activity of p56  **lck**   [ **LCK** ] .  Ref: 9858516 J Exp Med, 1998
- **Lck**  together with Itk was the best combination of kinases, effectively phosphorylating synthetic peptides corresponding to a cytoplasmic sequence of  **CD5** .  Ref: 12525577 J Leukoc Biol, 2003
- Using as a model an  **Lck**  mouse, we show that, while dispensable for B 1 cell development,  **Lck**  is required for full and sustained activation of the tyrosine phosphorylation and MAP kinase cascades triggered by the BCR in  **CD5** , B 1 cells.  Ref: 12645942 Eur J Immunol, 2003
- CD6 also associated with  **Lck** , Fyn, and ZAP 70, although at lower levels but additionally coprecipitated the Tec family kinase Itk, which is absent from CD2,  **CD5** , and TCR complexes.  Ref: 12525577 J Leukoc Biol, 2003

**Non-interactive relationship** (e.g. studied together, co-existance, homology, etc.)

- The  **CD5**  regulated substrates included CD3zeta, ZAP 70, Syk, and phospholipase Cgammal but not the Src family tyrosine kinase p56  **lck**   [ **LCK** ] .  Ref: 10082557 Mol Cell Biol, 1999
- The disappearance of T cells in the absence of Fyn and  **Lck**  was associated with a complete loss of TCRzeta chain phosphorylation and down regulation of  **CD5** , both of which are also MHC contact dependent, indicating that the Src family kinases are critical for transducing a TCR MHC survival signal.  Ref: 12218114 J Immunol, 2002
- We propose that T cell activation mediates  **CD5**  tyrosine phosphorylation at residues Y429 and Y463 mainly through the activation of  **Lck** .  Ref: 11298344 Eur J Immunol, 2001
- Using tyrosine phosphorylated peptides corresponding to  **CD5**  cytoplasmic sequences we also show that the Src homology 2 SH2 domain of p56  **lck**   [ **LCK** ]  binds prominently to pY429SQP, with 30 fold less affinity to pY463DLQ and not to pY441PAL.  Ref: 11157848 Int Immunol, 2001
